# Gene Network Analysis of Bone Marrow Mononuclear Cells Reveals Activation of Multiple Kinase Pathways in Human Systemic Lupus Erythematosus

**DOI:** 10.1371/journal.pone.0013351

**Published:** 2010-10-14

**Authors:** Magdalene Nakou, George Bertsias, Ilias Stagakis, Michael Centola, Ioannis Tassiulas, Maria Hatziapostolou, Iraklis Kritikos, George Goulielmos, Dimitrios T. Boumpas, Dimitrios Iliopoulos

**Affiliations:** 1 Division of Rheumatology, Clinical Immunology and Allergy, University of Crete Medical School, Heraklion, Greece; 2 Institute of Molecular Biology and Biotechnology, Foundation for Research and Technology, Heraklion, Greece; 3 Microarray Research Facility, Oklahoma Medical Research Foundation, Oklahoma City, Oklahoma, United States of America; 4 Department of Cancer Immunology and AIDS, Dana-Farber Cancer Institute, Boston, Massachusetts, United States of America; 5 Department of Pathology, Harvard Medical School, Boston, Massachusetts, United States of America; Centre de Recherche Public de la Santé, Luxembourg

## Abstract

**Background:**

Gene profiling studies provide important information for key molecules relevant to a disease but are less informative of protein-protein interactions, post-translational modifications and regulation by targeted subcellular localization. Integration of genomic data and construction of functional gene networks may provide additional insights into complex diseases such as systemic lupus erythematosus (SLE).

**Methodology/Principal Findings:**

We analyzed gene expression microarray data of bone marrow mononuclear cells (BMMCs) from 20 SLE patients (11 with active disease) and 10 controls. Gene networks were constructed using the bioinformatic tool Ingenuity Gene Network Analysis. In SLE patients, comparative analysis of BMMCs genes revealed a network with 19 central nodes as major gene regulators including ERK, JNK, and p38 MAP kinases, insulin, Ca^2+^ and STAT3. Comparison between active versus inactive SLE identified 30 central nodes associated with immune response, protein synthesis, and post-transcriptional modification. A high degree of identity between networks in active SLE and non-Hodgkin's lymphoma (NHL) patients was found, with overlapping central nodes including kinases (MAPK, ERK, JNK, PKC), transcription factors (NF-kappaB, STAT3), and insulin. In validation studies, western blot analysis in splenic B cells from 5-month-old NZB/NZW F1 lupus mice showed activation of STAT3, ITGB2, HSPB1, ERK, JNK, p38, and p32 kinases, and downregulation of FOXO3 and VDR compared to normal C57Bl/6 mice.

**Conclusions/Significance:**

Gene network analysis of lupus BMMCs identified central gene regulators implicated in disease pathogenesis which could represent targets of novel therapies in human SLE. The high similarity between active SLE and NHL networks provides a molecular basis for the reported association of the former with lymphoid malignancies.

## Introduction

Although gene profiling studies provide important information for key molecules relevant to a disease, they are less informative of protein-protein interactions, post-translational modifications and regulation by targeted subcellular localization. In several diseases, important proteins such as MAP kinases, are activated by phosphorylation while their mRNA and protein levels remain constant. To identify the molecular mechanisms by which these genes lead to complex disease phenotypes, such as obesity, diabetes, osteoarthritis, multiple sclerosis, juvenile rheumatoid arthritis and other autoimmunse diseases [1]–[4], it is essential to integrate genomic data and construct functional gene networks that will be predictive of these diseases.

By the use of microarrays in the peripheral blood or bone marrow, we and others have previously shown specific gene signatures that are involved in SLE and correlate with disease activity [5]–[8]. Compared to peripheral blood, bone marrow may be more informative in accessing immune reactions in SLE patients [5]. In our study, BM gene analysis differentiated SLE patients based on their disease activity and identified genes involved in apoptosis [5]. Accelerated apoptosis and impaired clearance of apoptotic cells due to the decreased phagocytic ability of macrophages, monocytes and neutrophils have a pathogenic role in SLE [9]–[11].

In this study, we identified the signaling networks where these genes are involved by integrating gene expression profiling data, derived from bone marrow of lupus patients and healthy individuals [5], using bioinformatic approaches. Identification of the central nodes (also called hubs) in these networks could uncover unique targets of novel therapies for lupus patients.

## Results

### Identification of gene network central nodes in SLE patients

**Figure 1:**
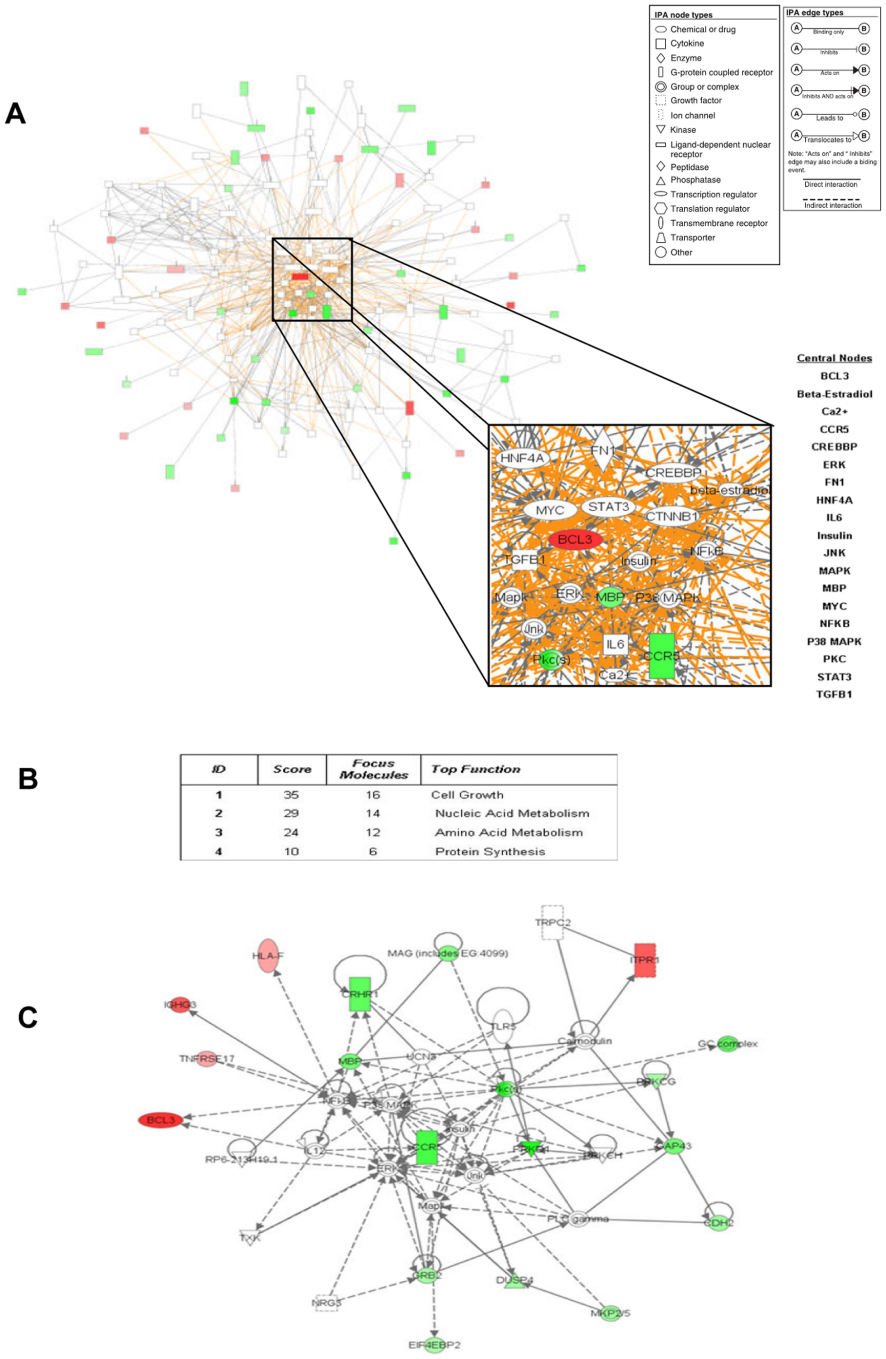
Pathway analysis of bone marrow genes in SLE patiens versus controls. (**A**) A gene network of 19 central nodes was constructed by using the differentially expressed genes in the bone marrow of SLE patients and controls, as described in *Materials and Methods*. Deletion of any of these nodes pertubates or destroys the gene network. Genes are colored according to gene expression value; red gene symbols indicate up-regulation and green gene symbols indicate down-regulation. Nodes are displayed using various shapes that represent the functional class of the gene product. Edges with dashed lines show indirect interaction, while a continuous line represents direct interactions (see explanatory inset). (**B**) Four gene sub-networks are related to nucleic and amino acid metabolism, cell growth and protein synthesis. A dataset containing the differentially expressed genes, called the *focus molecules*, between SLE and controls was overlaid onto a global molecular network developed from information contained in the Ingenuity Pathways Knowledge Base. Networks of these focus molecules were then algorithmically generated based on their connectivity. The composite *score* of the networks represents the negative log of the p-value for the likelihood that network molecules would be found together by chance. Accordingly, a higher score indicates greater statistical significance that molecules depicted in the network are interconnected. (**C**) The most significant gene network is involved in cellular growth.

Differentially expressed genes between SLE patients and controls were organized into an interactome network using Ingenuity Pathway Analysis (IPA). The differentially expressed genes derived from the microarray data constructed a gene network with 19 central nodes (including JNK, ERK, p38, Insulin, STAT3, FN1, Ca^2+^) (**Figure 1A**). These nodes are major gene regulators in the network as deletion of any of these nodes pertubates or destroys the network. This complex gene network consists of 4 major gene sub-networks (**Figure 1B**). The most significant gene network (*p* = 10^−35^) is involved in cellular growth and has as central nodes the following molecules: BCL3, JNK, insulin, p38 MAPK, MBP, PKC, NFκB, ERK, MAPK and CCR5 (**Figure 1C**). Among them, MBP, PKC, CCR5 were down-regulated (green color) in SLE, BCL3 was up-regulated (red color), while the remaining genes were not affected (white color) (Figure 1C).

### Identification of gene networks involved in disease activity

**Figure 2:**
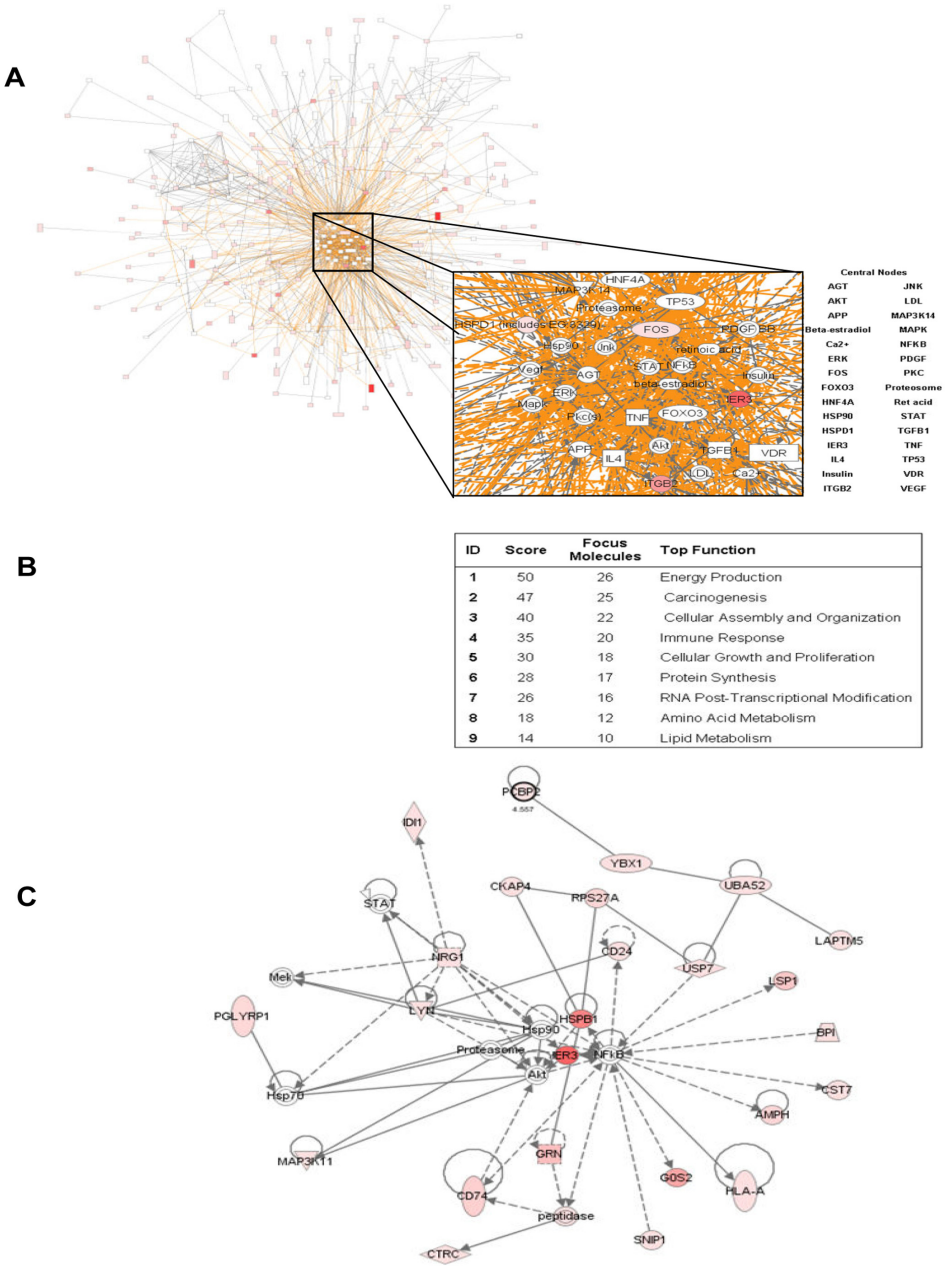
Network analysis of differentially expressed genes in the bone marrow of active versus inactive SLE patients. (**A**) Gene network analysis in the bone marrow of active versus inactive patients with SLE revealed 30 central nodes, and (**B**) 9 gene sub-networks that illustrate major functions of these pathways. (**C**) The most significant network consists of proteins that are up-regulated in active patients. See Figure 1 legend for more details on gene network analysis and description.

We next constructed gene networks based on the microarray analysis of active versus inactive SLE patients. **Figure 2A** shows 30 hubs (including ERK, STAT, AKT, PI3K, LYN, PDGF, FOS, TP53) consisting of 9 gene sub-networks (**Figure 2B**). The most significant gene network (*p* = 10^−50^) consisted mainly of proteins that were up-regulated in active patients (by our microarray analysis); AKT, NF-κB, HSP90, proteosome, IER3 and HSPB1 emerged as central nodes (**Figure 2C**). The identification of these proteins as central nodes supports their involvement in SLE activity.

### Correlation between lupus and other diseases on genomic level

To detect any possible correlation between lupus and other diseases at the genomic level, we compared the differentially expressed genes between SLE and control samples and other disease gene sets. Comparisons were made with the existing genomic data provided by the software. SLE was highly associated with neurological, renal, cardiovascular diseases and cancer (**Table S1**). When comparisons involved only those genes differentially expressed between SLE active and inactive patients, we found that gene networks from patients with increased activity were related to cancer, renal, infectious, and cardiovascular diseases (**Table S2)**.

Because of the high correlation between active SLE networks and cancer, we next sought to identify the types of cancer that correlated highly with lupus. Comparison literature-mining gene networks for the 18 most common types of cancer relative to lupus gene network, revealed a high similarity between SLE gene network derived from active SLE and non-Hodgkin's lymphoma (NHL) gene network. Specifically, we identified 23 hubs in the NHL gene network (**Figure S1A**), among which seven (ERK, JNK, MAPK, NFKB, PKC, STAT, insulin) were common with the SLE gene network **(Figure S1B)**.

### Activation of kinase pathways in lupus mice

**Figure 3:**
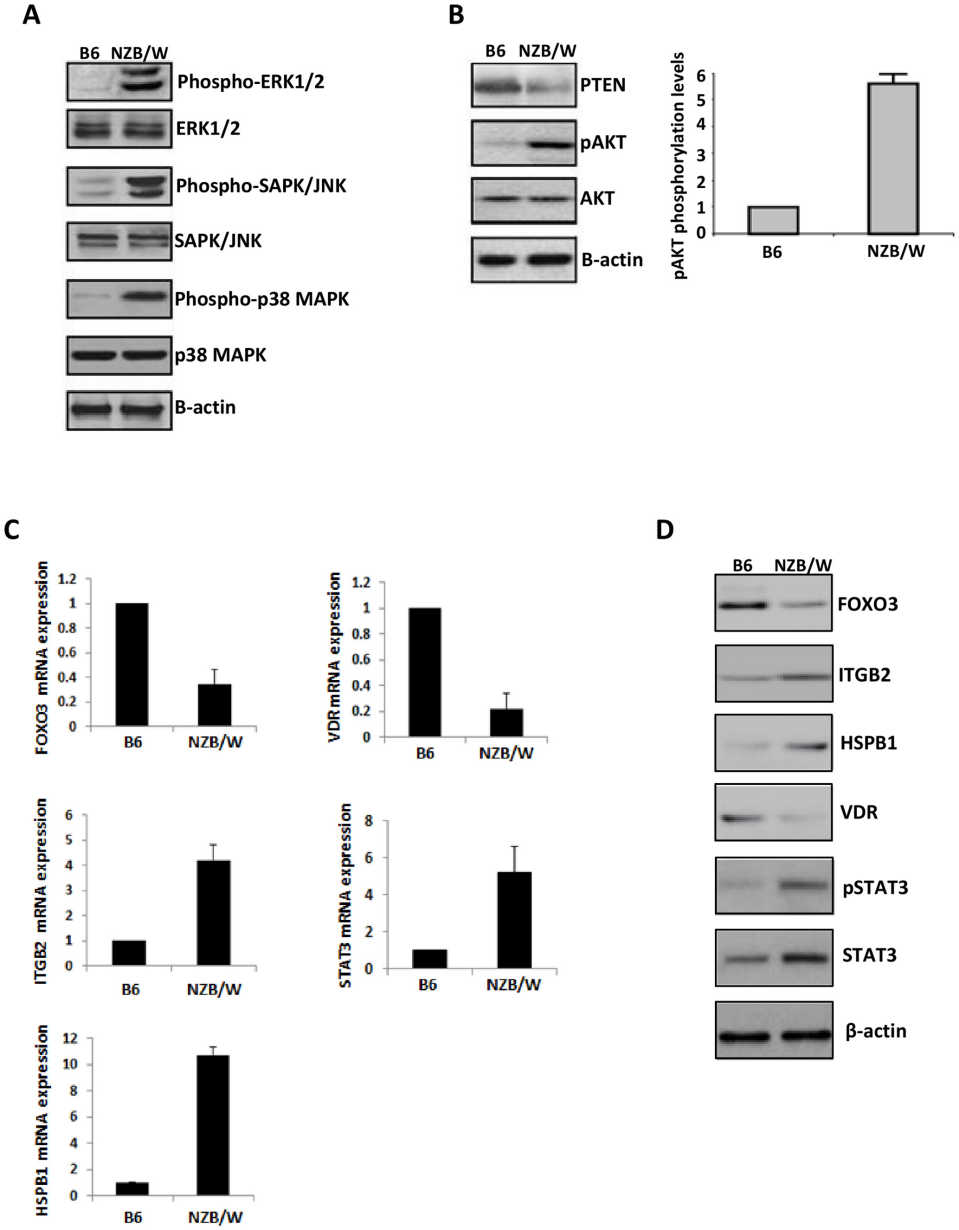
Validation of gene network results in NZB/NZW F1 lupus mice. (**A**) Western blot analysis in isolated spleen B cells from lupus NZB/NZW F1 mice revealed activation of several kinases identified in gene network analysis. ERK1/2, SAPK/JNK, and p38 MAPK kinases were phosphorylated while their total protein levels were not altered in NZB/NZW F1 compared to control C57Bl/6 (B6) mice. (**B**) Activation of AKT signaling pathway in NZB/NZW F1 mice. Quantitative real-time PCR (**C**) and western blot (**D**) analysis in isolated spleen B cells demonstrated downregulation of FOXO3 and VDR, and upregulation of STAT3, pSTAT3, ITGB2, and HSPB1 in NZB/NZW F1 compared to B6 mice.

Bone marrow is a central lymphoid organ with a major role in production, maturation and activation of B cells. To validate our findings from the gene network analysis, we examined the expression of several hubs identified from our analysis by quantitative real-time PCR and western blot analysis in spleen B cells obtained from C57Bl/6 and NZB/NZW F1 diseased mice. This analysis revealed a distinct set of kinases activated in NZB/NZW F1 mice. Specifically, ERK1/2, SAPK/JNK and p38 MAPK kinases were activated (phosphorylated) while their protein levels were not altered **(** **Figure 3A** **)**. We also found activation of the AKT signaling pathway, which is involved in cellular survival by inhibiting apoptotic processes. Specifically, the tumor suppressor gene PTEN was down-regulated whereas AKT phosphorylation was enhanced **(** **Figure 3B** **)**. Other gene network hubs that were validated in NZB/NZW F1 spleen B cells included the transcription factor FOXO3 and vitamin D receptor (VDR), which were both downregulated at mRNA and protein level **(** **Figures 3C, D** **)**. In contrast, ITGB2 (encoding the β2 integrin family), HSPB1 (co-chaperone that binds to and regulates the chaperone Hsp70), and STAT3 were upregulated. Western blot analysis further showed increased phosphorylation of STAT3 (pSTAT3) in lupus B cells **(** **Figure 3D** **)**.

## Discussion

SLE is a complex disease affecting multiple organs characterized by variable course and periods of remissions and flares. Although its etiology is not established, many pathogenic pathways are thought to contribute to tissue injury. Several of the central node molecules identified in our analysis have been implicated in lupus pathogenesis. Comparison between patients and controls predicted involvement of pathways related to both survival (ERK, JNK, MAPK, P38 MAPK, and BCL3) and immune-reactions (STAT3, NFKB, CCR5, MBP) in the development of SLE. The high similarity between active SLE and NHL gene networks is provocative in view of data documenting a correlation of SLE with malignancies, especially NHL where a 3 to 4-fold increased risk is observed [12], [13]. Our findings suggest that MAPK and inflammatory pathways link SLE with NHL at the molecular level.

Several human and murine studies that are not included in the IPA Knowledge Base® have demonstrated activation of multiple kinases and transcription factors including the MAPKs MEK1/ERK1/2, p38, the PI3K/AKT/mTOR axis, the NF-kB and multiple Bcl-2 family members, and the Jak/STAT pathway [14]–[19]. We confirmed activation of these kinases in B cells from NZB/NZW F1 lupus mice, predicted to play a central pathogenic role based on the gene network analysis. Activation of MAPKs, PI3K/AKT and NF-kB occurs downstream of receptors with a key role in recognition of antigens, immunoglobulins and nucleic acids, all of which have been linked to the aberrant immune response in lupus.

Our analysis also revealed calcium and STAT proteins as central regulators in SLE gene network [20]. An emerging aspect of the Jak/STAT signaling pathway biology is the cross-talk with ITAM-containing receptors and adaptor molecules and the cross-regulation by calcium-dependent signaling pathways. Increased levels and activation of STAT1 has been reported in the spleen, lymph nodes, and kidneys of MRL/lpr and NZB/NZW F1 lupus mice [21]. STAT1 phosphorylation in target organs was potentiated by type I interferons, which have a clearly defined pathogenic role in SLE. Moreover, pharmacologic inhibition of the calcium–dependent kinase CaMKII *in vivo* prevented IFNa-induced STAT1 phosphorylation and induction of pro-inflammatory genes in the target tissues of MRL/lpr and NZB/NZW F1 lupus mice. Increased phosphorylation of STAT5 and STAT3 have been reported in B cells of the congenic B6.*Sle1ab^z^* and *B6Sle1^z^.Sle3^z^* lupus mice [21], [22], and Harada *et al.* [23] have demonstrated increased STAT3 mRNA levels in T cells from SLE patients, associated with enhanced chemokine-induced cell migration. The presence of insulin as central node in SLE, confirms previous findings of changes in bioenergetics during T cell activation and systemic inflammation in humans. Insulin is required to support the elevated energetic and biosynthetic demands of growth proliferation and effector function [1], [24].

Comparison of active versus inactive SLE microarray data identified a total of 30 central nodes; in contrast comparison of SLE versus healthy controls revealed only 19 central nodes. This suggests that additional signaling pathways are deregulated in active SLE. Involvement of AKT, NF-κB, HSP90, proteosome, IER3 and HSPB1 as central nodes in the gene network of active patients highlights their importance in SLE since the PI3K/AKT/mTOR signaling pathway plays an important role in the differentiation of peripheral B cells and in T cell homeostasis. Over-expression of PI3K in T cells results in the development of lymphoproliferative and autoimmune disorders. Conversely, inhibition of PI3Kγ in the MRL/lpr mouse model of lupus reduced glomerulonephritis and prolonged life span [22], [25].

The strength of the gene network strategy was further documented by our validation experiments in lupus mice. Proteins such as ERK, JNK and p38 MAPK kinases, were not detected by microarray analysis as their mRNA levels were not altered. Nonetheless, they were predicted to be central molecules implicated in SLE pathogenesis based on integration of microarray data on differentially expressed genes and construction of gene networks. These data imply that several kinase pathways are activated in lupus and suggest the potential therapeutic use of kinase inhibitors in these patients.

Our validation analysis in NZB/NZW F1 B cells demonstrated decreased mRNA and protein levels of VDR, which mediates the pleiotropic immunological effects of vitamin D. Of note, several SLE cohorts have low vitamin D levels, and addition of vitamin D to PBMCs results in a significant reduction of polyclonal and anti-dsDNA antibody production by SLE B cells through direct inhibition of their differentiation [26]. In accordance with a previous study in lupus-prone (BXSB, MRL/lpr) mice [27], we also observed decreased expression of FOXO3, a transcription factor that participates in negative regulation of Th1 responses. Importantly, our human SLE gene network analysis and murine validation studies revealed – for the first time – a possible pathogenic role for: a) HSPB1, a co-chaperone that regulates the chaperone Hsp70 and is involved in stress-induced cell migration [28], and b) ITGB2, encoding the β2 integrin family, that was reported to protect against development of autoimmune diabetes in NOD/LtJ mice [29].

In summary, our analysis has revealed that lupus pathogenesis is contingent upon the activation of gene networks in which the pivotal nodes could be targets for development of new therapeutic strategies [30]. Gene network analysis based on gene profiling data may represent a powerful method to predict key gene regulators and identify shared pathways among distinct categories of complex diseases.

## Materials and Methods

### Patients

Gene expression (cDNA) microarray data were obtained from bone marrow mononuclear cells (BMMCs) from 20 SLE patients. Control sample included 7 healthy individuals and 3 patients with osteoarthritis (non-inflammatory knee arthritis). All patients met the 1982 American College of Rheumatology revised criteria for the classification of SLE [31] and were recruited from the Rheumatology Department, University Hospital of Heraklion (Greece). To capture patients with higher disease activity, we used a SLE Disease Activity Index (SLEDAI) score cut-off of ≥8; 11 patients were classified as active (5 patients had active proliferative and/or membranous nephritis while another 5 patients had active neuropsychiatric lupus with psychosis, major depression, myelitis, and polyneuropathy) and 9 as inactive. A written consent was obtained by all patients and healthy individuals and the study was approved by the Ethics Committee of the University Hospital of Heraklion.

### Generation of cDNA microarrays

Experimental procedures are described in detail elsewhere [5]. Briefly, BMMCs were isolated by Ficoll-Histopaque (Sigma-Aldrich, St. Louis, MO) density-gradient centrifugation of bone marrow aspirates, and were immediately placed in Trizol (Invitrogen, Carlsbad, CA, USA) and processed for RNA extraction using the RNeasy kit (Qiagen). cDNA was synthesized from 2 µg of RNA using Omniscript reverse transcriptase (Qiagen) with direct incorporation of Cy3-dUTP. Labeled cDNA was purified using Montage 96-well vacuum system (Millipore). cDNA was added to hybridization buffer containing human CoT-1 DNA (0.5 mg/ml), yeast tRNA (0.2 mg/ml), and poly(dA)_40–60_ (0.4 mg/ml).

Microarray production was performed using a commercially available genome-scale oligonucleotide library containing gene-specific 70-mer oligonucleotides and representing 21,329 human genes. The library includes 16 replicate spots of 12 random negative controls that are designed to have no significant homology to known human DNA sequences (Qiagen). Oligonucleotides were spotted onto Corning UltraGAPS™ amino-silane coated slides and were covalently fixed to the surface of the glass using 300 mJ of ultraviolet radiation at a 254 nm wavelength. Microarrays were scanned using a simultaneous dual-color, 48-slide scanner (Agilent Technologies).

### Microarray Expression Analyses

The R/Bioconductor Package “Affy” was used to perform gene expression quantile normalization to adjust the marginal distribution of each sample. Genes with average background adjusted fluorescent intensity value >50 across all arrays were retained in the analysis. The variance across genes was also calculated, and genes with a variance below the median value were considered unlikely to be differentially expressed (DE) and were not retained for further analysis. DE genes between two classes were identified through an unpaired Student's *t* test. A 10% false discovery rate *p*-value multiplicity adjustment was used. The false discovery rate is the proportion of the list of genes claimed to be DE that are false positives. Only statistically significant DE genes with greater than a 2-fold change in expression between groups were retained.

### Gene Network Analysis

Gene networks were constructed and important hubs were identified using Ingenuity Pathway Analysis (IPA; Ingenuity Systems, Mountain View, CA) based on DE genes between SLE patients and healthy individuals (*n* = 102 genes) and between active and inactive SLE (*n* = 245 genes) [5]. IPA is a robust and expertly curated database containing updated information on more than 20,000 mammalian genes and proteins, 1.4 million biological interactions, and 100 canonical pathways incorporating over 6,000 discreet gene concepts. This information is integrated with relevant databases such as Entrez-Gene and Gene Ontology. The experimental data sets were used to query the IPA and to compose a set of interactive networks taking into consideration canonical pathways, the relevant biological interactions, and the cellular and disease processes. Pathways of highly interconnected genes were identified by statistical likelihood using the following equation:
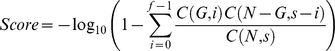



Where *N* is the number of genes in the network of which *G* are central node genes, for a pathway of *s* genes of which *f* are central node genes. *C(n,k)* is the binomial coefficient. We considered statistically significant networks those with a score greater than 5 (*p* value <10^−5^).

### Gene Ontology Analysis

Gene Ontology Analysis was performed using IPA. Specifically, we performed gene ontology analysis by using the lupus gene signatures (derived from comparisons between lupus versus control samples and also between lupus active versus inactive samples) and identified the correlation and statistical significance between lupus and any other disease. Gene sets were collected from previously published papers and included 1406 genes for various metabolic disorders [32], 494 genes for atherosclerosis [33], and 855 genes for malignant disorders [34]–[37]. We considered as statistical significant the correlations with a *p* value <0.05.

### Comparison between Lupus and Cancer Gene Networks

To correlate lupus with other diseases in the gene network level, we compared lupus gene network derived from our data with cancer (breast, lung, thyroid, gastric, colon, liver, pancreas, prostate, melanomas, Hodgkin lymphoma, non-Hodgkin's lymphoma, acute myeloid leukemia, chronic lymphocytic leukemia, acute lymphoblastic leukemia, chronic myelogenous leukemia) gene networks derived from the Ingenuity Program. The level of statistical significant (*p* value) was set to 10^−5^ to allow for multiple comparisons in gene networks.

### Protein Expression Analysis

Five-month old female NZB/NZW F1 mice were used. Mice were intercrossed and maintained in a temperature-controlled germ-free facility (Harvard Medical School, USA) on a 12-h light/dark cycle, and fed by standard chow diet and water *ad libitum*. Animal studies were performed according to National Institutes of Health (USA) guidelines following protocols approved by the Institutional Animal Care and Use Committee of Boston University (MA, USA). Spleen B cells were isolated by magnetic separation using CD19 microbeads (Miltenyi Biotec, Germany). Cells were lysed in RIPA buffer containing 50 mM Tris-HCl, pH 7.2, 150 mM sodium chloride, 1% NP-40, 12 mM sodium deoxycholate, 3 mM sodium dodecyl sulfate, 4 mM sodium azide, 0.57 mM phenylmethysulfonyl fluoride and 10 mg/L complete protease inhibitor coctail. Protein expression and phosphorylation levels of ERK1/2, SAPK/JNK, p38 MAPK and AKT proteins were evaluated by western blot analysis. The antibodies used for protein detection were: phospho-ERK1/2 (cat. no 4377, Cell Signaling), ERK1/2 (cat no. 4695, Cell Signaling), phospho-SAPK/JNK (cat no. 4668, Cell Signaling), SAPK-JNK (cat no. 9252, Cell Signaling), phospho-p38 MAPK (cat no. 4631, Cell Signaling), p38 MAPK (cat no. 9212, Cell Signaling), PTEN (cat no. sc-56205, Santa Cruz Biotechnology Inc), phospho-AKT S473 (cat no. sc-101629, Santa Cruz Biotechnology Inc), AKT (cat no. ab8805, Abcam Inc), FOXO3 (cat no. ab47409, Abcam), ITGB2 (cat no. ab86457, Abcam), HSPB1 (cat no. ab2790, Abcam), VDR (cat no. ab3508, Abcam), STAT3 (cat no. 9139, Cell Signaling), phospho-STAT3 (cat no. 9138, Cell Signaling), *β*-actin (cat no. ab8227, Abcam Inc).

### ELISA assay for assessing AKT S473 phosphorylation status

The phosphorylation status of Akt S473 was assessed by ELISA assay following the manufacturer instructions (cat.no SUV887, R&D Systems). This immunoassay employs a two-site sandwich ELISA to quantitate Akt phosphorylated at S473 in cell lysates. Spleen B cells were isolated by magnetic separation using CD19 microbeads (Miltenyi Biotec, Germany) and then were lysed on ice for 15 min and centrifuged at 2,000 g for 5 minutes. Supernatants were transferred into clean tubes and incubated with phospho-Akt antibody for 2 h at room temperature. After 3 washes the supernatants were incubated with streptavidin-HRP for 20 minutes at room temperature. The next step was to add 100 µl substrate solution and incubate for 20 minutes at room temperature followed by 50 µl of stop solution and then read at 450 nm within 30 minutes. Data represent the average of three different samples derived from different mice.

### mRNA Expression Analysis

Total RNA was extracted from spleen B cells by the Trizol method followed by RNeasy columns purification. Equal amounts of purified RNA samples from NZB/NZW F1 and control C57Bl/6 mice were reverse-transcribed to cDNA, which was subjected to SYBR Green based real-time polymerase chain reaction analysis. The following sets of primers were used: FOXO3: 5′-CAACCAAGGAAATGCTCCTC-3′ (forward) and 5′-ACAAAGTGAGCCGTTTGTCC -3′ (reverse), ITGB2: 5′-ACCAGCCCAGAGGTGACTGT-3′ (forward) and 5′-CTGCTCCTGGATGCACTCTGT -3′ (reverse), HSPB1: 5′-GCCGCACCAGCCTTCAGC-3′ (forward) and 5′-CACGCCTTCCTTGGTCTTCACT-3′ (reverse), VDR: 5′-CTGACCCTGGAGACTTTGAC-3′ (forward) and 5′-TTCCTCTGCACTTCCTCATC-3′ (reverse), STAT3: 5′-AAGGAGACATGCAGGATCTGA -3′ (forward) and 5′-TTCTGCACGTACTCCATTGC-3′ (reverse), β-actin: 5′-GAGACCTTCAACACCCCAGC-3′ (forward) and 5′-ATGTCACGCACGATTTCCC-3′ (reverse). The amplification efficiencies of the target and the reference genes were similar as indicated by the standard curves, and the following formula was used: fold difference  = 2^−(ΔCtA–ΔCtB)^, where Ct is the cycle threshold.

## Supporting Information

Table S1 Differentially expressed genes between SLE patients and controls highly correlate with other diseases on genomic level.
(0.03 MB DOC)

Table S2 Differentially expressed genes between active and inactive SLE patients correlate with other diseases on genomic level.
(0.03 MB DOC)

Figure S1 Gene network analysis identifies a high degree of identity between active SLE and non-Hodgkin's lymphoma patients. (A) Gene networks in patients with non-Hodgkin's lymphoma (NHL). (B) Overlapping central nodes between SLE and NHL gene networks.
(2.26 MB TIF)
