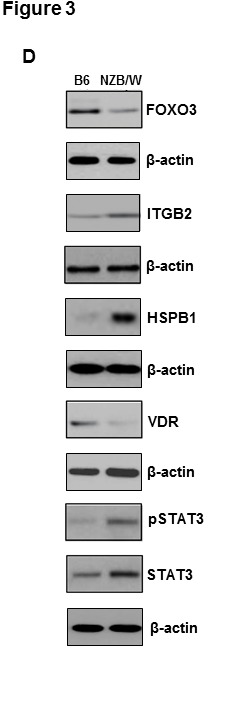# Correction: Gene Network Analysis of Bone Marrow Mononuclear Cells Reveals Activation of Multiple Kinase Pathways in Human Systemic Lupus Erythematosus

**DOI:** 10.1371/annotation/d68d4b94-c44a-4893-83f4-55a8a626990f

**Published:** 2013-04-10

**Authors:** Magdalene Nakou, George Bertsias, Ilias Stagakis, Michael Centola, Ioannis Tassiulas, Maria Hatziapostolou, Iraklis Kritikos, George Goulielmos, Dimitrios T. Boumpas, Dimitrios Iliopoulos

Since Figure 3D is composed of proteins that originate from five separate blots, we are adding the relevant β-actin controls for each of the blot available in the corrected figure. In addition, in the same figure, the panel for HSPB1 protein has been replaced with a replicate HSPB1 panel due to the fact that we were not able to retrieve the original blot. All the data are consistent with the results in the published article.

**Figure:**