# Supplementary material for: Gene Network Analysis of Bone Marrow Mononuclear Cells Reveals Activation of Multiple Kinase Pathways in Human Systemic Lupus Erythematosus
Source: PLoS One. 2010 Oct 14;5(10):e13351. doi: 10.1371/journal.pone.0013351 (PMC2954787; doi:10.1371/journal.pone.0013351)
Supplement: Table S1 — Differentially expressed genes between SLE patients and controls highly correlate with other diseases on genomic level. (0.03 MB DOC) [file pone.0013351.s001.doc]

**Table S1. Differentially expressed genes between SLE patients and controls highly correlate with other diseases on genomic level.**

| **Group of diseases** | ***P* value ^1^** |
| --- | --- |
| Neurological diseases | 2.35×10^-6^ |
| Renal – urological diseases | 4.22×10^-4^ |
| Cardiovascular diseases | 5.88×10^-4^ |
| Malignancies | 3.98×10^-3^ |

^1^ Level of statistical significance for the correlation between SLE patients and patients with the indicated diseases on genomic level (see *Materials and Methods* for more details).
