# Supplementary material for: Gene Network Analysis of Bone Marrow Mononuclear Cells Reveals Activation of Multiple Kinase Pathways in Human Systemic Lupus Erythematosus
Source: PLoS One. 2010 Oct 14;5(10):e13351. doi: 10.1371/journal.pone.0013351 (PMC2954787; doi:10.1371/journal.pone.0013351)
Supplement: Table S2 — Differentially expressed genes between active and inactive SLE patients correlate with other diseases on genomic level. (0.03 MB DOC) [file pone.0013351.s002.doc]

**Table S2. Differentially expressed genes between active and inactive SLE patients correlate with other diseases on genomic level.**

| **Group of diseases** | ***P* value ^1^** |
| --- | --- |
| Malignancies | 7.27×10^-7^ |
| Renal – urological diseases | 4.22×10^-6^ |
| Infectious diseases | 3.67×10^-4^ |
| Cardiovascular diseases | 5.15×10^-4^ |

^1^ Level of statistical significance for the correlation between active SLE patients and patients with the indicated diseases on genomic level (see *Materials and Methods* for more details).
