# Supplementary figures and images for: Gene Network Analysis of Bone Marrow Mononuclear Cells Reveals Activation of Multiple Kinase Pathways in Human Systemic Lupus Erythematosus
Source: PLoS One. 2010 Oct 14;5(10):e13351. doi: 10.1371/journal.pone.0013351 (PMC2954787; doi:10.1371/journal.pone.0013351)

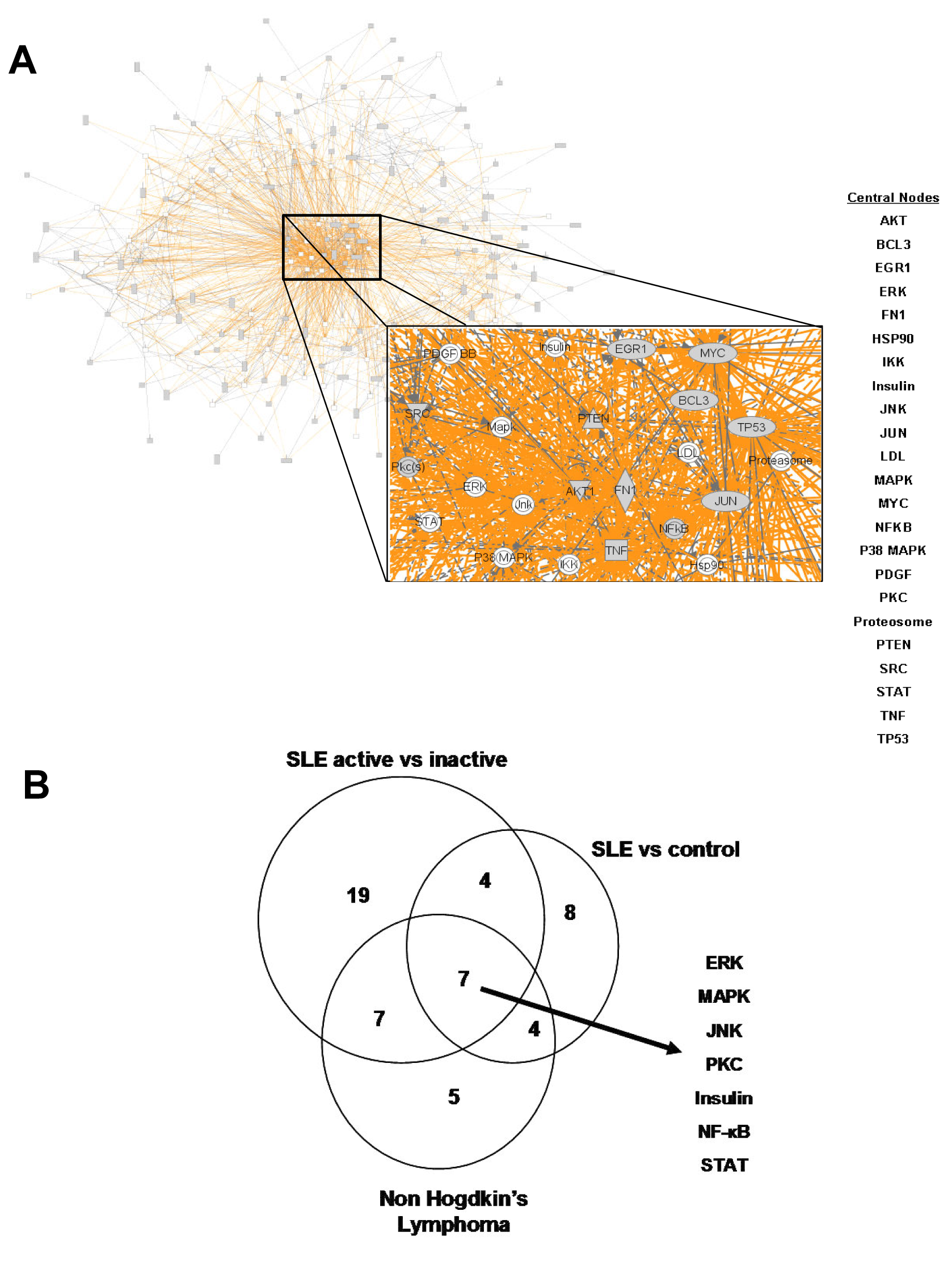

Supplement: Figure S1 — Gene network analysis identifies a high degree of identity between active SLE and non-Hodgkin's lymphoma patients. (A) Gene networks in patients with non-Hodgkin's lymphoma (NHL). (B) Overlapping central nodes between SLE and NHL gene networks. (2.26 MB TIF) [file pone.0013351.s003.tif]
